# Three-dimensional quantitative analysis of healthy foot shape: a proof of concept study

**DOI:** 10.1186/s13047-018-0251-8

**Published:** 2018-03-09

**Authors:** Kristina Stanković, Brian G. Booth, Femke Danckaers, Fien Burg, Philippe Vermaelen, Saartje Duerinck, Jan Sijbers, Toon Huysmans

**Affiliations:** 1 imec – Vision Lab, Dept. of Physics, University of Antwerp, Universiteitsplein 1, Antwerp, B-2610 Belgium; 2 RSscan International, De Weven 7, Paal, 3583 Belgium; 3 Runners’ Lab, Westpoort 68, Zwijndrecht, 2070 Belgium; 4 Section on Applied Ergonomics & Design, Department of Industrial Design, Delft University of Technology, Landbergstraat 15, CE Delft, 2628 The Netherlands

**Keywords:** 3D foot shape, Statistical modelling, Subject characteristics, Foot loading, Foot asymmetry

## Abstract

**Background:**

Foot morphology has received increasing attention from both biomechanics researches and footwear manufacturers. Usually, the morphology of the foot is quantified by 2D footprints. However, footprint quantification ignores the foot’s vertical dimension and hence, does not allow accurate quantification of complex 3D foot shape.

**Methods:**

The shape variation of healthy 3D feet in a population of 31 adult women and 31 adult men who live in Belgium was studied using geometric morphometric methods. The effect of different factors such as sex, age, shoe size, frequency of sport activity, Body Mass Index (BMI), foot asymmetry, and foot loading on foot shape was investigated. Correlation between these factors and foot shape was examined using multivariate linear regression.

**Results:**

The complex nature of a foot’s 3D shape leads to high variability in healthy populations. After normalizing for scale, the major axes of variation in foot morphology are (in order of decreasing variance): arch height, combined ball width and inter-toe distance, global foot width, hallux bone orientation (valgus-varus), foot type (e.g. Egyptian, Greek), and midfoot width. These first six modes of variation capture 92.59% of the total shape variation. Higher BMI results in increased ankle width, Achilles tendon width, heel width and a thicker forefoot along the dorsoplantar axis. Age was found to be associated with heel width, Achilles tendon width, toe height and hallux orientation. A bigger shoe size was found to be associated with a narrow Achilles tendon, a hallux varus, a narrow heel, heel expansion along the posterior direction, and a lower arch compared to smaller shoe size. Sex was found to be associated with differences in ankle width, Achilles tendon width, and heel width. Frequency of sport activity was associated with Achilles tendon width and toe height.

**Conclusion:**

A detailed analysis of the 3D foot shape, allowed by geometric morphometrics, provides insights in foot variations in three dimensions that can not be obtained from 2D footprints. These insights could be applied in various scientific disciplines, including orthotics and shoe design.

## Background

Human foot morphology is an important subject for physical anatomical analysis in several biomedical disciplines, including orthopedics, orthotic design and sports sciences [1–13]. Different environments and everyday habits (e.g., frequency of sport activity, shoe wearing habits), as well as personal characteristics such as sex, body mass index, and age, have been shown to have a significant influence on adult foot morphology [1–9]. Human foot shape also differs among ethnic groups [2] and changes in the course of postnatal development [10]. As a result, footprint shape has been used in a variety of disciplines such as orthopedics [11, 12], and footwear research [13].

A common approach to study foot morphology is to analyze the two-dimensional footprint, despite the potential loss of information along the vertical dimension [1, 14–16]. The reason for the ubiquitous use of footprints is that they can be relatively easily obtained, measured, and preserved by using wax, plaster, foam or dynamic pressure plates [17–20]. To fill the missing 3D shape information along the vertical dimension, feet tend to be classified into discrete types, such as pes planus (flat foot) and pes cavus (high-arched foot), by visual inspection of footprint shape [10, 14, 15]. A wide range of different quantitative measures and indices of footprint shape, mainly based on the geometry of the medial longitudinal arch, have also been proposed [14]. Based on these parameters, various foot typologies have been defined [10, 14, 16]. Most of these quantifications are based on a small number of footprint shape characteristics, such as the sizes of different footprint regions, the curvature of the medial longitudinal arch, or the orientation of the forefoot relative to the rearfoot [14, 15].

Nonetheless, these quantitative measures are insufficient to describe the entire 3D foot shape. The study of Luximon et al. [21] showed that generating 3D foot shape from 2D information for custom footwear design introduces error in the 3D foot shape, revealing that there is additional information in 3D shape compared to 2D footprint. Overall, a 3D foot scanner is recommended for collecting foot anthropometric data because it has relatively high precision, accuracy and robustness [22]. A promising technique to examine this full 3D shape information is statistical shape modelling. This technique is used in dysmorphology training [23] and various product design applications [24]. Statistical shape modelling has also been successfully employed in foot classification [25] based on metatarsal bones geometry, but only a partial 3D foot shape is described (i.e. the position of the metatarsal bones).

To date, statistical modelling of the full 3D foot shape has yet to be achieved. Such a model could be beneficial in various applications. In clinical examinations, a statistical model of healthy 3D foot shape could be used as a baseline to which a patient’s 3D foot scan can be compared. In footwear design, a 3D foot shape model could help produce footwear with a better accommodation for foot girth.

In the present paper, we propose a methodology to quantify the 3D shape of whole feet based on geometric morphometrics, which is a standard technique used for the analysis of 3D shapes in biological datasets [25–27]. We employ geometric morphometrics on anatomically matched 3D meshes of feet from a healthy population. The aligned meshes preserve foot topology, and therefore statistical results, such as group means or principal components, describe actual foot shapes and foot shape deformations. Using geometric morphometrics, we examine the healthy 3D foot shape, the bilateral asymmetry of foot shape, and the difference in shape between different foot loadings. The influence of personal characteristics (e.g. body mass index, sex, age, frequency of sport activity) on the foot shape are also investigated.

## Methods

### Data collection

Our cohort contains 62 adults equally split between males and females. The Ethics Committee of the Antwerp University Hospital approved the study and all subjects gave their written informed consent before participating.

All individuals were considered to have healthy feet if they had never been diagnosed with foot pathology or injury requiring medical intervention, had no foot complaints (i.e. no foot pain), and no incidental findings were found at the time of data collection, as evaluated by a physical therapist. In particular, the height of the foot arch was not an excluding factor for our cohort, so the individuals with certain ranges of the height of the arch, which are considered to be normal, were selected for our cohort. In fact, 13 (20.97%) individuals were considered to have high arched feet, 7 (11.30%) individuals were considered to have flat feet, while 42 (67.73%) individuals were considered to have normal foot arch.

Foot breadth diagonal and foot length [22] were determined by applying the Principal Component Analysis (PCA) to vertices which belong to plantar surface of each foot. In this way, we obtained 3 main axes of variation for each plantar surface. Once we have the main axes of variation, we determined the foot length as the difference between the maximum and minimum value along the first axis. Similarly, the foot breadth was computed as the difference between the maximum and minimum value along the second axis. The shoe sizes for each sex are distributed as follows and are given using both the European and Mondopoint scales. Mondopoint scale: The range for female shoe size was [224/90, 278/105], with average shoe size 246/93 (±14/5), while the range for male shoe size was [248/93, 291/109], with average shoe size 271/103 (±8/4). European scale: The range for female shoe size was [36.8, 45.7], with average shoe size 39.8 (±2.2), while the range for male shoe size was [40.6, 47.6], with average shoe size 43.9 (±1.6).

Additionally, demographic information was collected for the cohort (Table 1). All factors except shoe size were self-reported. We note that significant group differences were found for shoe size between sex (*t* = -17.138, *p* <0.001). Also, a significant correlation was found between body mass index and age (*ρ* = 0.35, *p* <0.001).

**Table 1:** Cohort demographics

	Age[years]	Shoe size [European(Mondopoint)]	Weight[kg]	Height[cm]	BMI	Frequency of sport activity[hours/week]
*μ*	38.9	40.9 (258/98)	72.6	175.0	23.7	3.7
*σ*	13.5	2.2 (17/6.8)	12.0	9.3	3.5	4.1
min	18.0	36.0 (224/90)	53.0	156.0	18.6	0.0
max	60.0	46.0 (291/109)	107.0	196.0	35.8	15.0

The 3D foot scans were acquired with an Elinvision FootIn3D laser 3D foot scanner (rs scan, Belgium). The 3D accuracy of the scanner is 0.3*mm*, while the mesh resolution is 3.02*mm*. A total of four scans were made for each person, two of the left foot (half loaded: bearing 50% of body weight, and full loaded: bearing 100% of body weight) and two of the right foot (half loaded and full loaded). Before scanning of the full loaded foot starts, the participant is allowed to establish their balance by holding a side wall and setting their free leg in the most comfortable position. This position is held during approximately 15 s required to obtain the scan. The scans of left feet were mirrored to the coordinate system of the right feet. Prior to the analysis, all 3D scans were cropped just above the ankle (lateral malleolus) to decrease the effect of different ankle poses obtained from half loaded and full loaded scans. Once cropped, a 3D mesh was triangulated [28] and the obtained 3D mesh was used for further analysis.

### Methods

#### Geometric morphometry

**Fig. 1:**
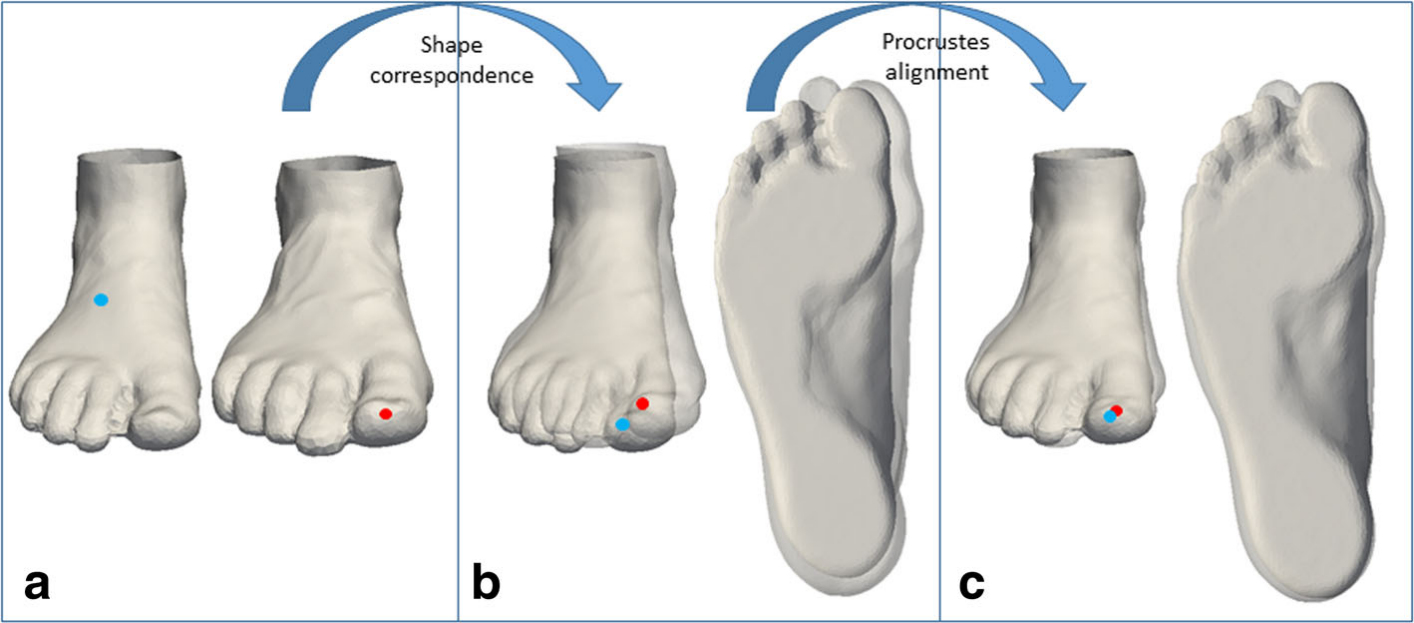
Example of shape correspondence and Procrustes alignment. **a** Two randomly chosen foot meshes. Initially, the vertices (e.g. blue and red points) on these meshes do not correspond; **b** Foot meshes after shape correspondence. Their vertices (e.g. blue and red points) are matched and located on the same anatomical position; **c** Foot meshes after Procrustes alignment. The geometric distance between corresponding vertices (e.g. blue and red points) is minimized

Before the shape variation in the population can be statistically analyzed, the 3D foot meshes need to be brought into correspondence and superimposed (Fig. 1a). This is done in two steps. First, mesh vertices are matched across subjects based on their anatomical similarity. Then, those matches are used to bring all feet into anatomical alignment (Fig. 1).

##### Shape Correspondence:

Initially, the vertices in our 3D meshes are randomly ordered, meaning that, say, vertex 511 in one foot mesh does not anatomically correspond to vertex 511 in another foot mesh (Fig. 1a). The number of vertices may also be different for every mesh. Before performing statistical analysis on these meshes, we must first establish an anatomical correspondence between them. To do so, we choose one foot mesh, *X*_*reference*_, as our reference foot and deform it to match the other feet in the database. This deformation is described by

1 $$ X_{target} = \Psi(T(X_{reference}), \beta)  $$

where *X*_*target*_ is a foot mesh in the database, *T* is an affine transformation (which rotates, shifts, and scales the whole foot mesh), and *Ψ* is an elastic deformation operation. The degree of the deformation operation is controlled by a user-defined elasticity parameter *β*. We solve for *T* and *Ψ* using the iterative procedure defined in [29]. Briefly, this iterative procedure operates by keeping one of the unknowns fixed (e.g. *Ψ*) and then solving Eq. 1 for the other unknown (e.g. *T*). Subsequently, the procedure solves Eq. 1 for the other unknown (*Ψ*) by keeping the previously-computed unknown (*T*) fixed. This iterative process repeats until no changes are observed in either *Ψ* or *T*. During these repetitions, the elasticity, *β*, is increased to gradually introduce more deformation as the alignment improves. Further details can be found in [29]. The final result was that the reference surface *X*_*reference*_ is deformed to have its shape as similar as possible to the shape of the target surface *X*_*target*_. At this point, *X*_*target*_ is replaced by *Ψ*(*T*(*X*_*reference*_),*β*), ensuring that each foot mesh has the same number of vertices ordered in the same fashion. This consistent vertex order ensures that every foot mesh has the same vertices in the same anatomical positions (Fig. 1b).

##### Procrustes Alignment:

Once shape correspondence has been established across all 3D foot meshes, the meshes still need to be brought into spatial alignment before statistics can be accurately performed. To obtain this alignment, all meshes are superimposed by a Generalized Procrustes Analysis [30]. This analysis consists of three steps that normalize the 3D foot meshes for position, size, and orientation (Fig. 1c). In the first step, all meshes are translated to have the same centroid (average vertex position). Next, the meshes are scaled to have the same size. In the last step, the meshes are rotated to minimize the summed squared distances between the vertices and their corresponding sample average. The above procedure is followed for each individual. To avoid reference bias, the whole approach is iterated three times, where in each iteration, the population average calculated from the previous iteration is used as the reference foot [31].

We performed a PCA of the aligned mesh vertices to investigate the major components of variation in 3D foot shape and to determine the mean 3D shape. PCA models each 3D mesh as follows:

2 $$ X=M+\sum_{i=1}^{n}P_{i}w_{i}  $$

where *X* is the 3D shape, *M* is the mean 3D shape, *P*_*i*_ is the *i*^th^ principal component (PC) and *w*_*i*_ represents the contribution of that PC in the shape. The first PC captures most of the population’s shape variance. For each individual, a score (*w*_*i*_) along the PC can be computed. The following PCs are computed to be uncorrelated to previous PCs, while also explaining as much of the remaining subject variation as possible. A single PCA was performed on the whole cohort, including left and right feet, males and females, and different foot loadings.

#### Statistical analysis

To assess the influence of different subject factors on 3D foot shape, we applied multivariate linear regression between the factors and their most relevant PCs:

3 $$  F=WB+E  $$

where *F* is the factors matrix, *W* is the matrix of the principal component contributions of each population member for the most relevant PCs (i.e. the *w*_*i*_ values from Eq. 1), *B* is the matrix of regression coefficients, and *E* ∼*N*(0,*σ*^2^*I*). The recorded cohort demographics of sex (1=male, 0=female), age, shoe size, BMI, frequency of sport activity, foot loading (1=half loaded, 0=full loaded), and foot side (1=right foot, 0=left foot) were selected as factors. The subset of relevant PCs, *W*, was determined through dimensionality reduction by sequential forward selection [32]. Bayesian information criterion (BIC) was used to select PCs and to determine when to stop the dimensionality reduction. In this way, only the PCs that best predict the subject factors are used for the statistical analysis. A statistical power analysis (*post hoc*) was applied to the obtained results.

#### Foot shape changes as a result of different foot side or loading

Finally, we also employed geometric morphometrics and multivariate linear regression to investigate how subject characteristics impact foot asymmetry and loading. To examine the correlation between foot asymmetry and other factors, a non-binary asymmetry measure was included. First, both right and reflected left foot were brought into correspondence and aligned. Then, we defined the foot asymmetry measure as the Euclidean distance between each vertex on the right foot and the corresponding vertex on the left foot. Next, we performed PCA on the obtained foot asymmetry measure (vectors containing all vertex distances) and applied multivariate linear regression of the PCs on the remaining factors. We further employed a non-binary loading measure between half and full loaded feet, in the same manner as for determining the asymmetry measure (i.e. using the Euclidean distance between feet under different loads). Finally, to examine the correlation between different foot loading and other factors, we performed the above PCA and linear regression procedures in the same manner as for the asymmetry measure.

## Results

### Principal component analysis

**Fig. 2:**
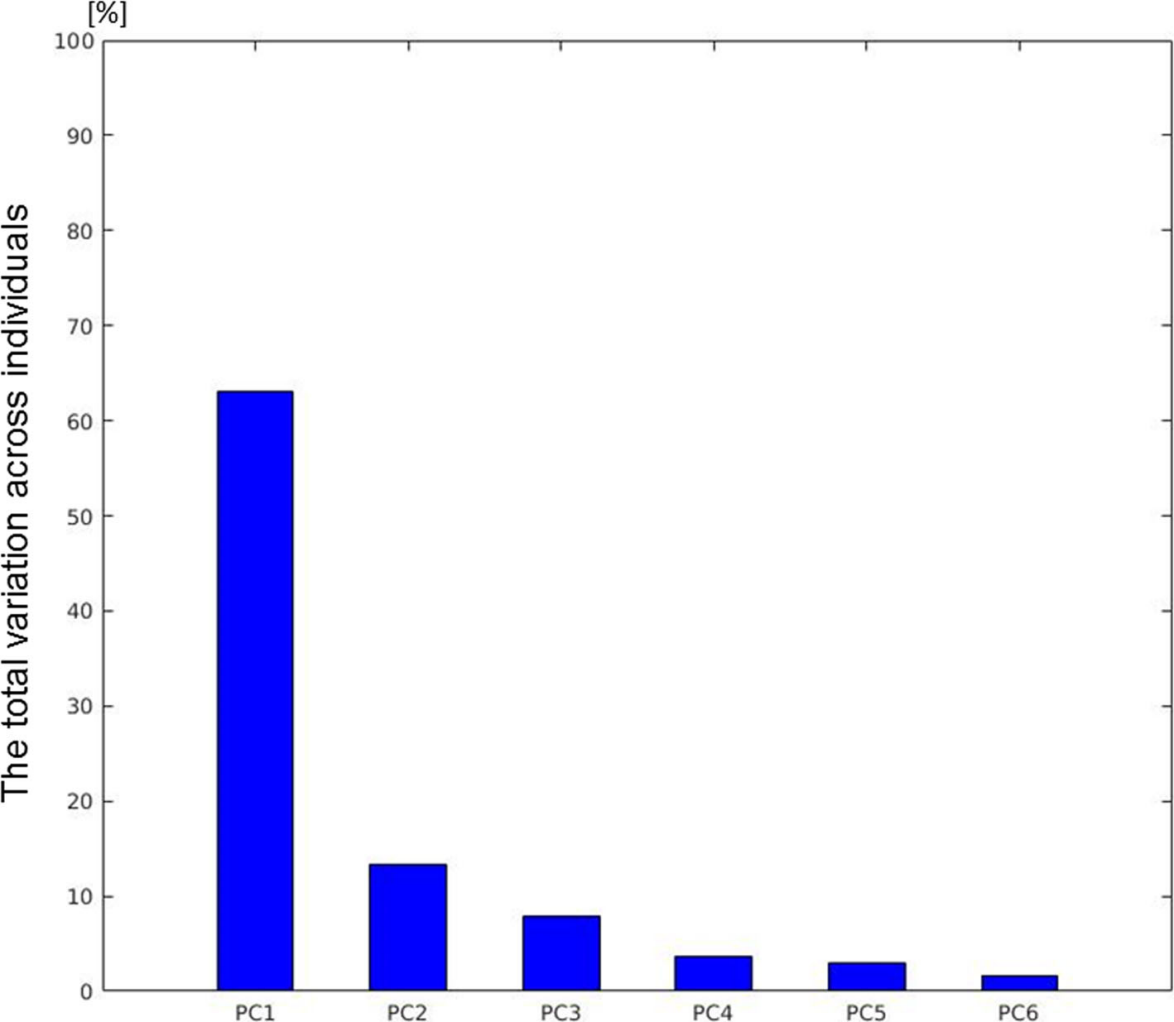
The amount of between-subject variance in 3D foot shape described by the first 6 principal components (PCs)

**Fig. 3:**
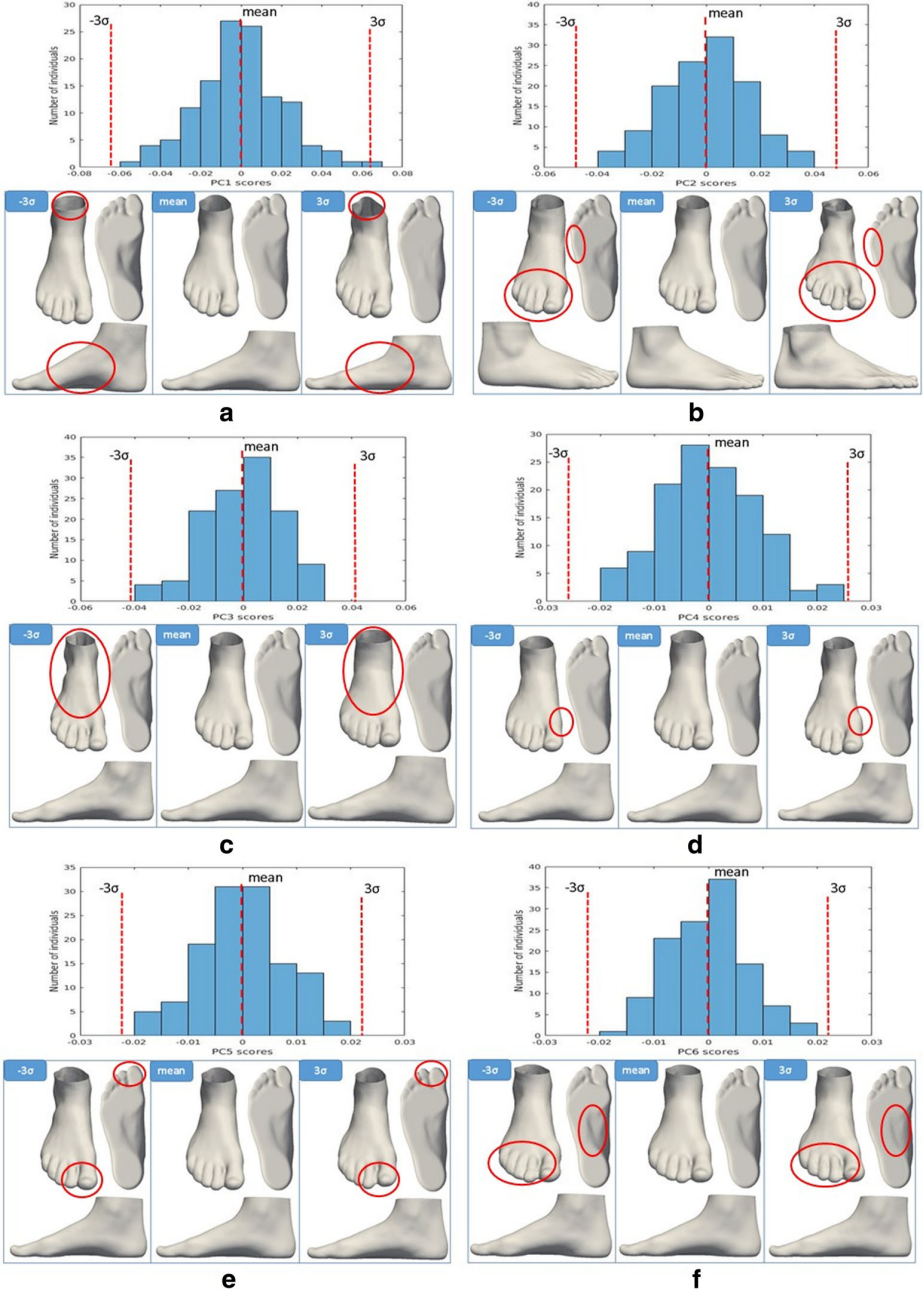
Histograms and views of the first six PCs of the 3D foot shape in a healthy population. **a** The first principal component (visualized by the foot shapes along the PC 1 axis) is a contrast between high arched feet (low PC 1 sores) and flat feet (high PC 1 scores). **b** The second principal component (visualized by the foot shapes along the PC 2 axis) represents the differences between narrow ball width with touching toes (low PC 2 scores) and wide ball width with spread out toes (high PC 2 scores). **c** The third principal component (visualized by the foot shapes along the PC 3 axis) is a contrast between narrow feet (low PC 3 sores) and wide feet (high PC 3 scores). **d** The fourth principal component (visualized by the foot shapes along the PC 4 axis) represents the differences between feet with normal hallux bone (low PC 4 scores) and feet whose hallux bone is angled towards the other toes-hallux valgus (high PC 4 scores). **e** The fifth principal component (visualized by the foot shapes along the PC 5 axis) is a contrast between “Egyptian” foot type (low PC 5 sores) and “Greek” foot type (high PC 5 scores). **f** The sixth principal component (visualized by the foot shapes along the PC 6 axis) represents the differences between feet with a narrow midfoot with toes angled laterally (low PC 6 scores) and feet with a wide midfoot with toes angled medially (high PC 6 scores)

Figure 2 shows the first six PCs, in order of decreasing variance, explaining 92.59% of the total foot shape variation. The shape variations identified by the first 6 PCs are shown in Fig. 3. Each PC is interpreted as a deformation of the mean foot shape, and is shown by adding (+3 *σ*) and subtracting (-3 *σ*) it from the mean foot shape (Fig. 3). The given size of each PC (32,272 numbers per PC) represents a limitation when displaying them. To illustrate the variation captured by each PC, we display its effect on the mean shape in Fig. 3 and marked what we observed. We noted that PC 1 principally captures the variation between high arched feet (pes cavus: low PC 1 score) and flat feet (pes planus:high PC 1 score) as well as the extent of Achilles tendon protrusion. We observed that PC 2 mainly captures the variation between feet with a narrow ball width (low PC 2 score) and feet with a wide ball width (high PC 2 score). Moreover, a low PC 2 score appears to characterize feet with small distance between toes, while a high PC 2 score appears to characterize feet with spread out toes. We detected that PC 3 mostly captures the variation in global foot width, including the ankle width, as well as variation in ball/waist/instep girth. We noted that PC 4 chiefly reflects the variation in the position of the hallux bone; individuals with high scores along PC 4 had feet with the hallux valgus, while individuals with low scores had feet with a hallux varus. We observed that PC 5 mostly represents variation in the shape of the toes and in the ankle angle. Individuals with a low score on this component are referred to as having Egyptian feet compared to individuals with high score who are referred to as having Greek feet. Individuals with Egyptian feet have the hallux as the longest toe, while individuals with Greek feet have a longer second toe [33]. We detected that PC 6 mainly reflects variation in midfoot width and direction of toes. Proportionally, the differences in variation of the latter PCs (PC 4, PC 6) are not that large since the shape variations they represent are more localized and limited in range in healthy populations. A larger variation along these PCs might be observed in a population including patients with, for example, a pathological degree of hallux valgus.

### The influence of factors on the foot shape

We investigated the influence of different subject factors on foot shape by regressing the principal component weights on the respective variables. For all factors, after dimensionality reduction, the 28 most relevant PCs were retained. Table 2 shows the statistical significance (*α* = 0.05) of the factors. The obtained R-squared values (Table 2) sum to a value greater than one, which shows that each factor describes a high percentage of variation within the model, and that the same shape variations occur as a result of multiple factors. This was expected as we had already found significant correlation between factors. We noted a significant correlation between age and BMI (*ρ* = 0.35, *p* <0.001), and significant differences in shoe size due to sex (*t* = -17.138, *p* <0.001). A *post hoc* statistical power analysis was also performed on each subject factor. The results of this analysis are given for each factor in Table 2. The factors of sex, age, shoe size, frequency of sport activity, and BMI all had significant influence on 3D foot shape (*p* <0.05) and high statistical power (> 0.8).

**Table 2:** Statistical significance of the linear relation between subjects factors and foot shape

	Sex	Age	Shoe size	Frequency of sport activity	BMI	Foot asymmetry	Foot loading
R-squared	0.9034	0.5959	0.8490	0.4042	0.7363	0.3566	0.3689
*p*	< 0.001	< 0.001	< 0.001	< 0.001	< 0.001	< 0.001	< 0.001
*post hoc* statistical power	1.0	0.9992	1.0	0.8640	1.0	0.7614	0.7905

We examined the correlation between each factor and first six PCs. The obtained results are shown in Table 3. As a result of our linear regression analysis, a factor’s strong positive correlation with a certain PC could be balanced out by its possible strong negative correlation with another PC. Therefore, each individual correlation cannot be interpreted independently. Instead, the impact of a subject factor on the foot shape should be examined as a whole.

**Table 3:** Correlation between subjects factors and first six PCs

	Sex *ρ* (*p*)	Age *ρ* (*p*)	Shoe size *ρ* (*p*)	Frequency of sport activity *ρ* (*p*)	BMI *ρ* (*p*)	Foot asymmetry *ρ* (*p*)	Foot loading *ρ* (*p*)
PC1	0.72*(0.001)	-0.019 (0.761)	0.60*(< 0.001)	0.19*(0.002)	0.0574 (0.368)	-0.0253 (0.691)	-0.26*(< 0.001)
PC2	-0.33*(< 0.001)	0.2246*(< 0.001)	-0.23*(0.002)	-0.0452 (0.478)	0.16*(0.011)	0.045 (0.480)	0.14*(0.024)
PC3	0.0113 (0.860)	-0.122 (0.054)	0.17*(0.008)	0.24*(< 0.001)	-0.53*(< 0.001)	0.0871 (0.171)	0.06578 (0.302)
PC4	-0.067 (0.291)	-0.1164 (0.067)	0.00789 (0.901)	0.0174 (0.785)	-0.13*(0.033)	0.08831 (0.166)	0.26*(< 0.001)
PC5	0.01847 (0.772)	-0.0192 (0.764)	-0.0363 (0.570)	-0.0887 (0.164)	0.17*(0.007)	0.14*(0.025)	0.06364 (0.318)
PC6	0.0018 (0.978)	-0.0068 (0.915)	-0.06156 (0.334)	-0.23*(< 0.001)	0.01258 (0.844)	-0.24*(< 0.001)	0.05232 (0.412)

^*^Significant at the 0.05 probability level

**Fig. 4:**
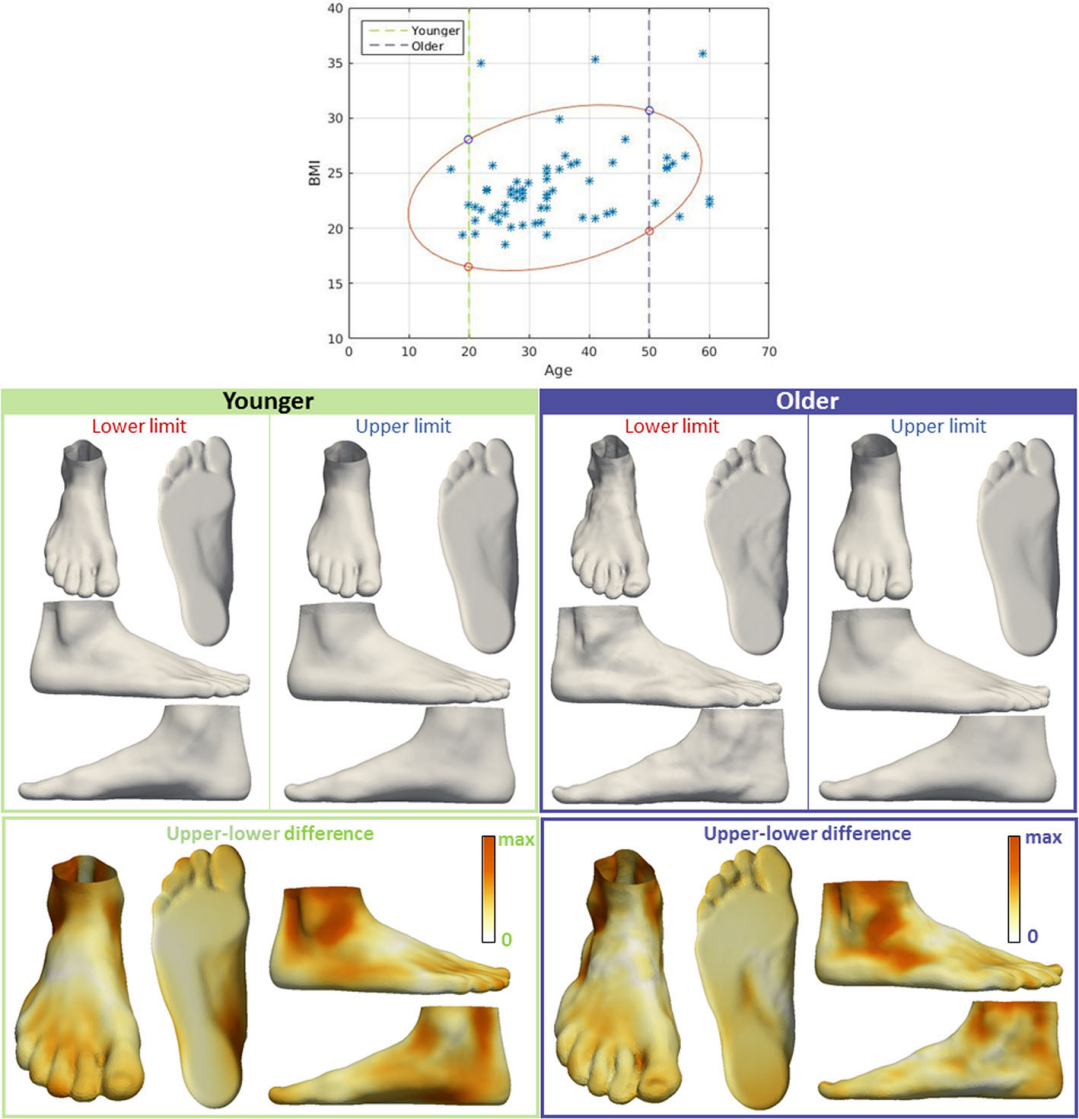
Visualization of the effect of BMI on foot shape. The influence of BMI on foot shape of younger people (20 years old, green box), and the influence of BMI on foot shape of older people (50 years old, purple box). Upper and lower limits are determined for each group as intersections with contour that covers the range that 90% of the values fall into. For each group, the influence of BMI is represented by color-mapped Euclidean distance computed between the foot shape obtained for upper limit and the foot shape obtained for lower limit

**Fig. 5:**
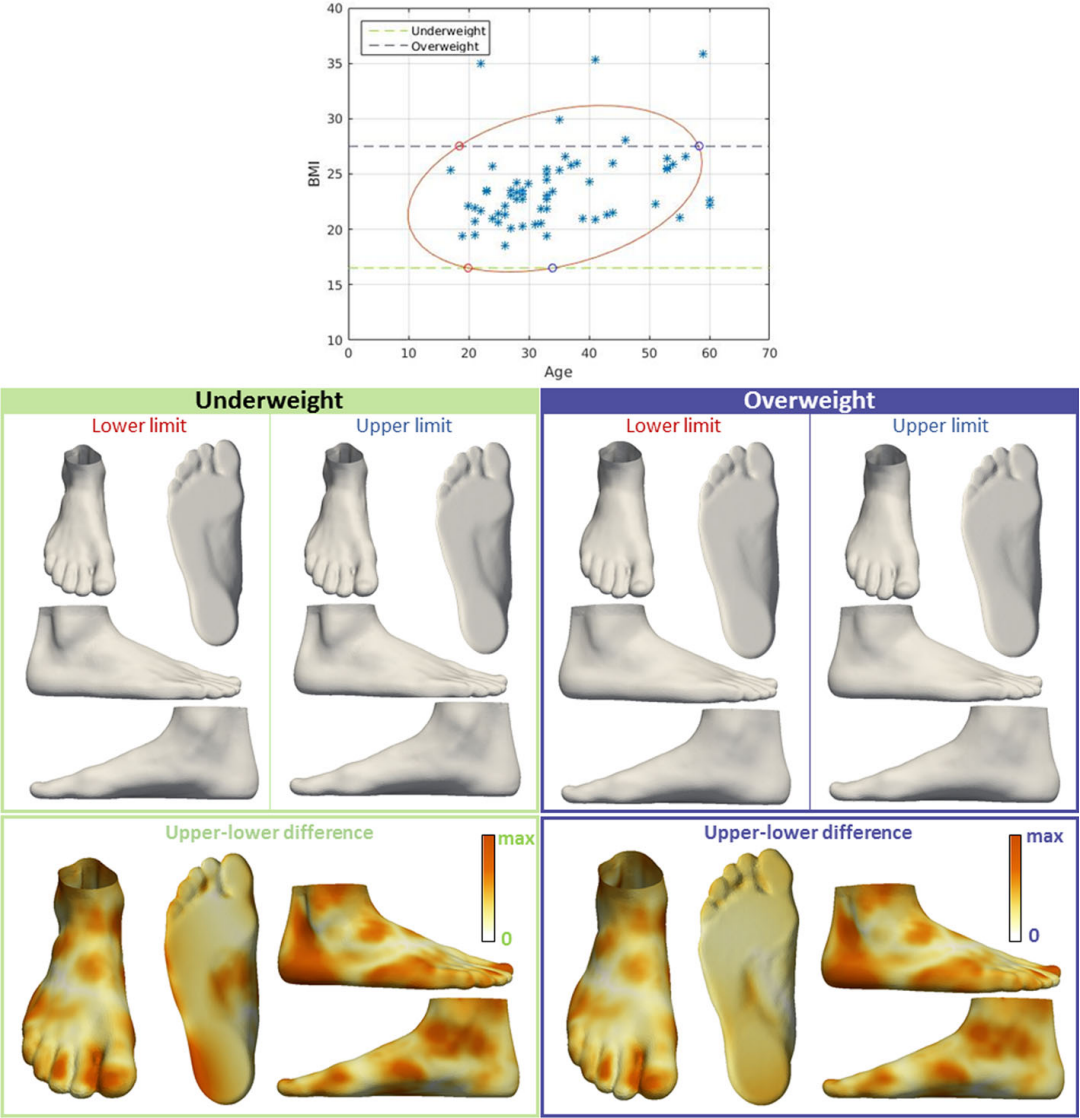
Visualization of the effect of age on foot shape. The influence of age on foot shape of underweight people (16.5 BMI, green box), and the influence of age on foot shape of overweight people (27.5 BMI, purple box). Upper and lower limits are determined for each group as intersections with contour that covers the range that 90% of the values fall into. For each group, the influence of age is represented by color-mapped Euclidean distance computed between the foot shape obtained for upper limit and the foot shape obtained for lower limit

**Fig. 6:**
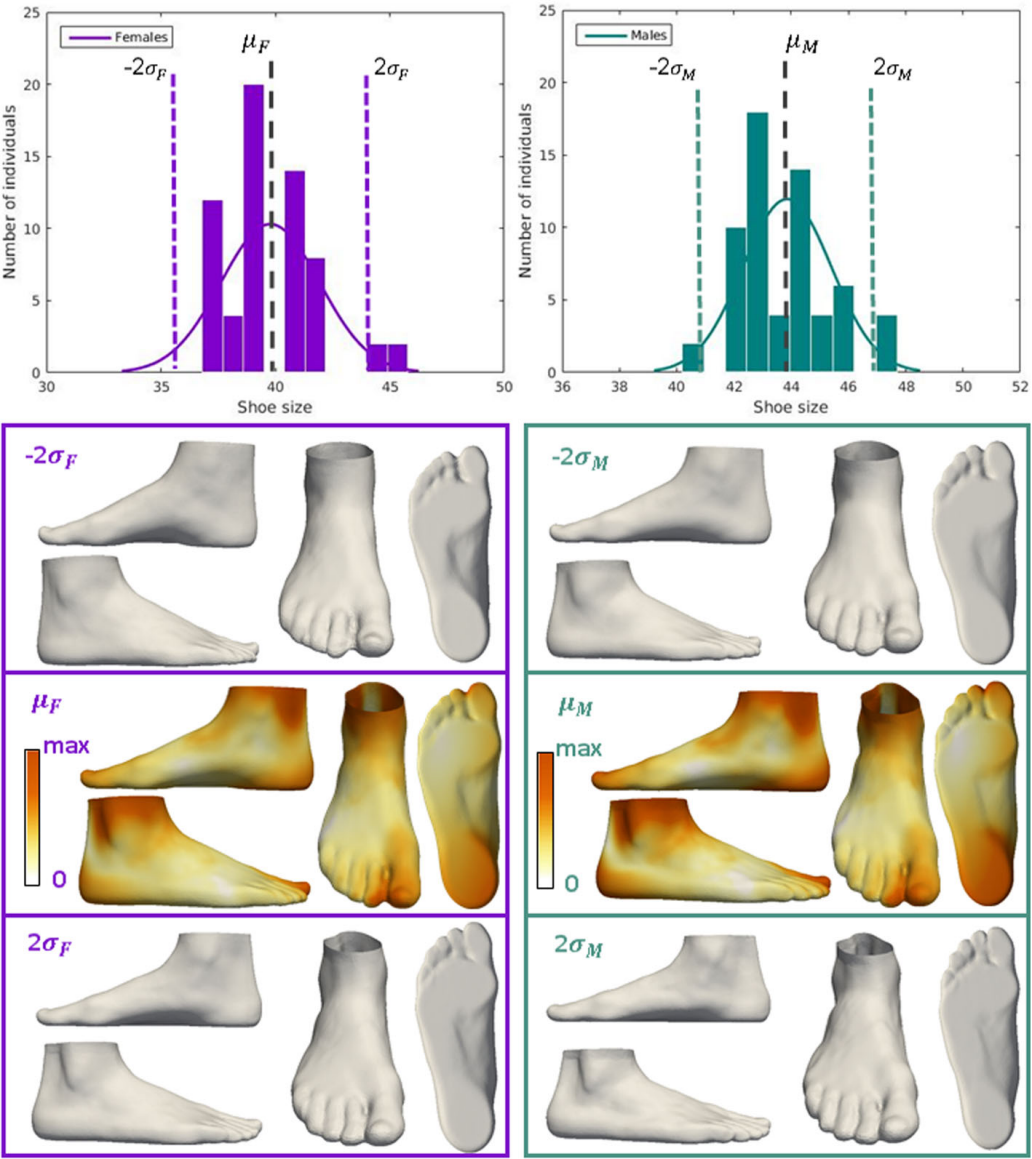
Visualization of the effect of shoe size on foot shape. The influence of shoe size on foot shape for females (purple box), and the influence of shoe size on foot shape for males (green box). For each group, the influence of shoe size is represented by color-mapped Euclidean distance computed between the foot shape obtained for *μ*+2 *σ* and the foot shape obtained for *μ*- 2*σ*

**Fig. 7:**
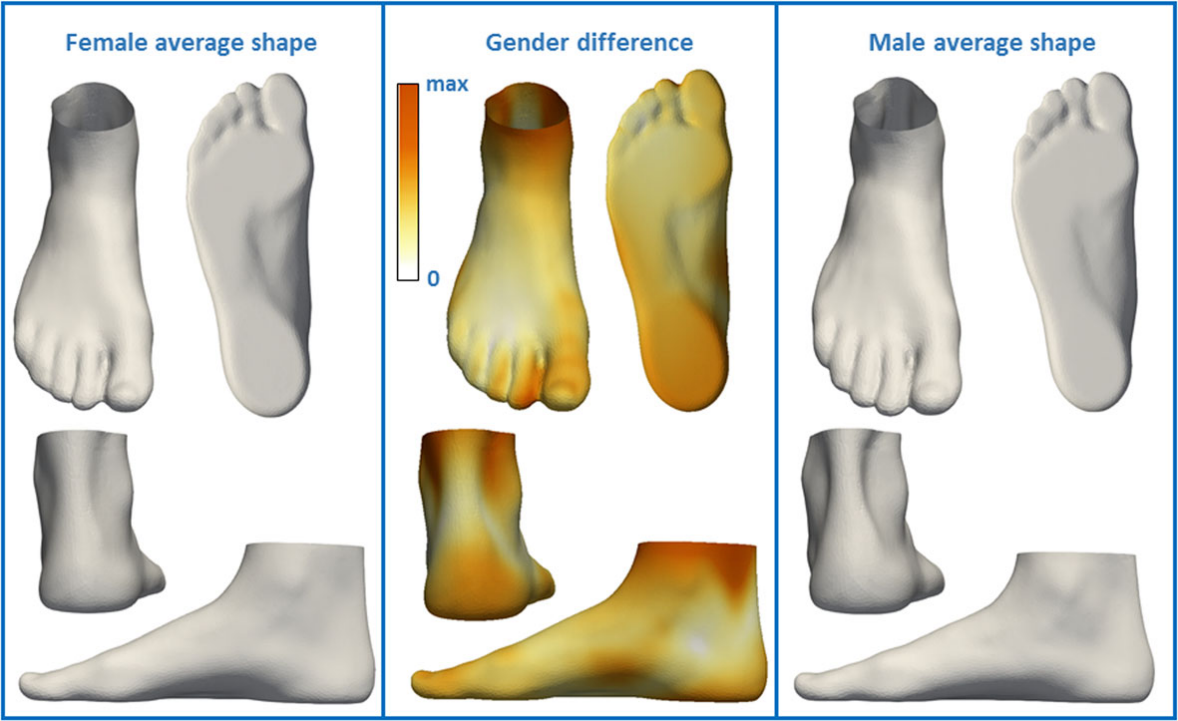
Visualization of the effect of sex on foot shape. The influence of sex is represented by color-mapped Euclidean distance computed between average male foot shape and average female foot shape

**Fig. 8:**
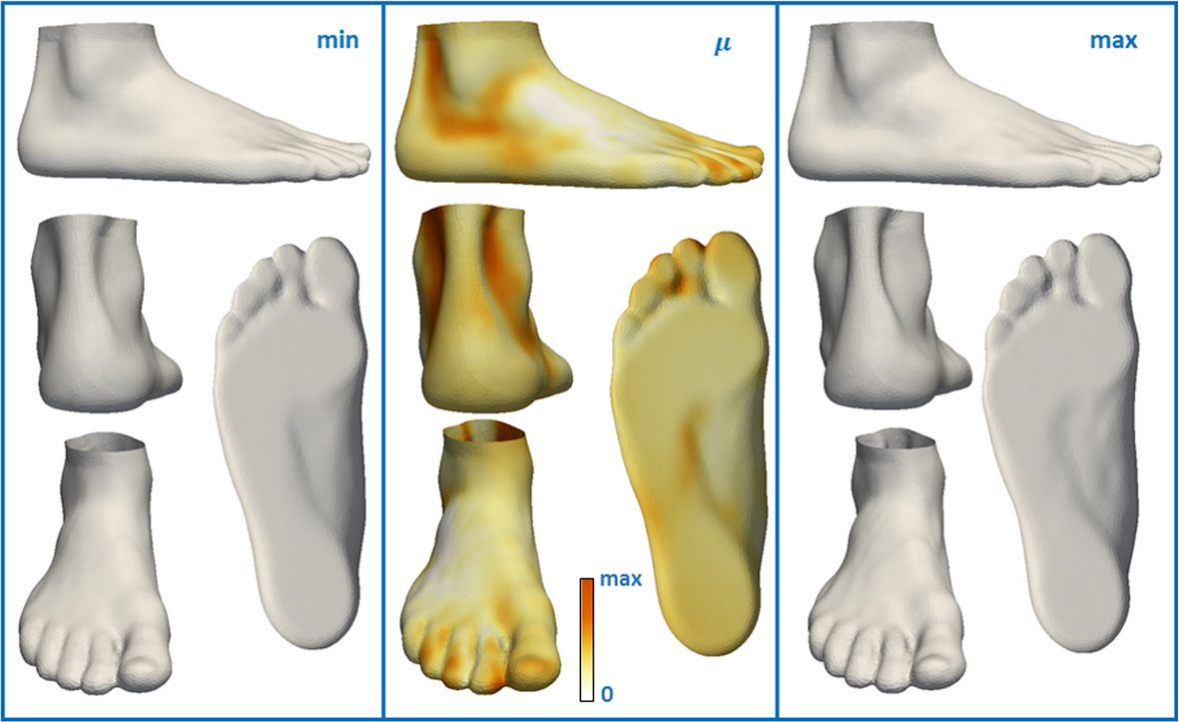
Visualization of the effect of frequency of sport activity on foot shape. The influence of frequency of sport activity on foot shape (middle), the expected foot shape for 0 hours/week (left), the expected foot shape for 15 hours/week (right). The middle picture represents color-mapped Euclidean distance computed between the foot shape obtained for maximum frequency of sport activity and the foot shape obtained for minimum frequency of sport activity

To visualize the impact of subject factors on foot shape, we used our linear regression model to predict foot shapes when varying each factor in isolation. The representative feet shown in Figs. 4, 5, 6, 7 and 8 were quantitatively derived from the linear model in Eq. 2. The changes in foot shape were observed when varying the corresponding subject factor in Eq. 2, while other factors remained fixed (to their averages, or in the case of correlations between factors, to the values shown in the figures). Our evaluation of these shape variations are described below.

First, we examined the relationship between BMI and foot shape (Fig. 4) by fixing the age for two groups: younger and older people (both age groups are examined due to the correlation between age and BMI). We observed that a low BMI was associated with a narrow ankle, a narrow Achilles tendon region, a narrow heel, a narrow midfoot, a straight heel in the sagittal plane, and a thinner forefoot along the dorsoplantar axis. We found that midfoot width, ball girth, waist girth and instep girth all increase with BMI. Similarly, we investigated the influence of age on foot shape (Fig. 5) by fixing the BMI for two groups: underweight and overweight. We noted that younger individuals were associated with a more noticeable Achilles tendon, a hallux varus, a more noticeable cuboid bone, a wider midfoot, and a narrow heel.

Next, we investigated the relation between shoe size and foot shape (Fig. 6) for males and females (both sex were examined separately due to their significant difference in shoe size). We observed that a smaller shoe size was associated with a wider Achilles tendon, hallux valgus, a wider heel, a straight heel in the sagittal plane, and a higher arch. We found a significant influence of sex on foot shape (Fig. 7). We noted that female foot has a narrower ankle width, a hallux valgus, a narrower Achilles tendon, a higher arch, and a narrower heel compared to the male foot.

Finally, we studied the influence of the frequency of sport activity on foot shape. We found that a low frequency of sport activity is associated with a wider Achilles tendon, a wider midfoot, and smaller toe height (Fig. 8).

Additionally, we have investigated the influence of foot asymmetry and foot loading on foot shape, by involving the binary factors for left/right feet and full/half loaded feet. Although the obtained *p* for foot asymmetry and foot loading are less than 0.05, the statistical power of those results are smaller than 0.8. Due to these low statistical powers, we cannot conclude that foot asymmetry and loading have a significant influence on foot shape (Table 2).

### Correlation between factors and non-binary foot asymmetry/foot loading

Finally, we examined the correlation between foot asymmetry and other factors, and foot loading and other factors. Neither foot asymmetry nor foot loading were associated to any of the remaining factors.

## Discussion and conclusion

We applied geometric morphometric methods to study 3D variations in foot shape on a database of 3D foot scans collected from healthy adults. Geometric morphometricics was previously used to study the variation of foot shape based on footprints [1]. We expanded on that research by capturing shape variation in the vertical dimension. We further investigated the influence of several factors on foot shape.

Our results showed similar foot shape phenomena as has been reported in previous studies of 2D footprints. These findings include a high BMI being associated with wide and flat feet [1, 2, 7], shoe size having a significant influence on foot shape [1], and significant differences being found between sex in arch height, Achilles tendon width, and hallux angle [8]. Our reported variations in foot shape with age also match previous literature [9, 34], and our lack of significant relationships regarding foot asymmetry, and foot loading match what has been previously reported on 2D footprints [35, 36].

The performed analysis on 3D foot shape revealed the advantages in using 3D shape compared to 2D, showing the foot shape variation in three dimensions. For example, the first PC of 3D foot shape (representing the major axes of variation) captured low-arched versus high-arched feet, as well as showing significant variation in the mediolateral position of the foot arch (Fig. 3a). High-arched feet tend to have the midfoot moved medially compared to low-arched feet. The heel and Achilles tendon become more noticeable for flat feet (Fig. 3a). The distances between toes and the ball width change along PC2. PC2 showed that feet with spread out toes have a wider ball width, compared to the feet that have smaller distance between toes (Fig. 3b). A notable difference in ankle and heel width between narrow and wide feet (Fig. 3c), as well as the difference in ball/waist/instep girth size are revealed along PC3. The variation in orientation of the hallux bone (PC4) showed that the cuboid bone becomes more noticeable when the hallux bone is angled medially (hallux varus). These results could not have been found with previous 2D footprint analysis.

The analysis that examines the influence of the factors on foot shape, revealed some information about the vertical variations visible only for 3D foot shape. Higher BMI results in a thicker forefoot along the dorsoplantar axis, a wider Achilles tendon, a wider heel, and a wider ankle which can be seen only in the 3D foot scan (Fig. 4). In particular, the 3D foot shape revealed that older people tend to have a wider heel, a less noticeable Achilles tendon, but also the hallux valgus, and higher toes compared to younger people (Fig. 5). The difference in ankle width, Achilles tendon width, and heel width between males and females is also distinct in the 3D foot shape (Figs. 6 and 7). The influence of shoe size on foot shape showed that a bigger shoe size was associated with narrow Achilles tendon, hallux varus, a narrow heel, extended heel in posterior direction, and a lower arch. People that are more physically active tend to have a more narrow Achilles tendon, a more narrow midfoot, and higher toes (Fig. 8). To the best of our knowledge, these results were not previously observed.

Despite the advantages of 3D methods over 2D, our approach has some limitations. Three-dimensional analysis of the foot shape, requires the input of 3D foot scans and hence, the availability of a 3D scanner. This is a notable disadvantage over 2D footprint analysis methods. Additionally, the findings presented herein were observed on a cohort of only 62 individuals. These individuals were also all adults and therefore did not cover the full range of mature foot shapes [37]. Finally, it should be noted that ethnicity was not considered as part of this study. As a result of these constraints, it is possible that the shape variability described here is not a complete representation of the possible 3D foot shapes present in a healthy population. Despite this limitation, we do show that our 3D foot shape model captures morphological information not present in a 2D footprint model. Furthermore, the theoretical properties of geometric morphometrics have been well-studied and the results we have shown fall well within the range of what is reliable for this analysis technique [38, 39].

Our findings could prove valuable in various areas of application. For example, they could allow footwear manufacturers to adapt the 3D design of a shoe based on how a customer’s factors influence their 3D foot shape. Our results show that the current approach of creating the shoes based only on 2D footprints [10] does not fully capture the wide variability in foot morphology. As a result, the quality of a shoe fit could be improved upon by using the comprehensive 3D foot shape in shoe design.

Our findings on 3D foot shape might also prove useful in routine clinical examinations. Our statistical analyses on healthy individuals could possibly aid in further standardizing and automating clinical evaluation. A recent study by Knapik on injury risk based on the plantar surface shape came to a similar conclusion [13]. In the study of Knapik, there tended to be a bimodal relationship between BMI and injury risk among the men. Our findings showed that the 3D foot shape changes as BMI changes. Therefore, taking this relationship into consideration during future research could potentially result in decreasing the foot injury risk. Relatedly, the study of Billis et al. [12] emphasizes the importance in everyday clinical practice to use more than one assessment technique of foot posture. Usage of the 3D foot shape during such a clinical examination might give more insight into the relationships between different foot parts, thereby improving diagnosis. These are just a few areas that could benefit from the reported findings.

In summary, healthy 3D foot shape, which we quantified using geometric morphometrics, gives more insight of the complex shape of the foot as compared to 2D footprint analysis. We found that the personal characteristics of sex, age, BMI, frequency of sport activity and shoe size, all significantly influence the 3D foot shape. This information about healthy 3D foot shape has the potential to be used for various purposes within several biomedical disciplines, including in the design of more accurate footwear and in the facilitation of more objective clinical diagnosis techniques. Our future work will be focused on extending our results from this study to these two areas of application.

## Abbreviations

BMI: Body mass index
PC: Principal component
PCA: Principal component analysis
